# Functional Activity Limitation and Quality of Life of Leprosy Cases in an Endemic Area in Northeastern Brazil

**DOI:** 10.1371/journal.pntd.0003900

**Published:** 2015-07-01

**Authors:** Victor S. Santos, Laudice S. Oliveira, Fabrícia D. N. Castro, Vanessa T. Gois-Santos, Ligia M. D. Lemos, Maria do C. O. Ribeiro, Luis E. Cuevas, Ricardo Q. Gurgel

**Affiliations:** 1 Postgraduate Program in Health Sciences, Federal University of Sergipe, Aracaju, Sergipe, Brazil; 2 Department of Nursing, Federal University of Sergipe, Aracaju, Sergipe, Brazil; 3 Liverpool School of Tropical Medicine, Liverpool, United Kingdom; University of Tennessee, UNITED STATES

## Abstract

**Background:**

Few studies have evaluated the association between quality of life (QoL) and functional activity limitations (FAL) of leprosy patients as determined by the Screening of Activity Limitation and Safety Awareness scale (SALSA).

**Aim:**

To identify the association between FALs and the QoL of patients during and post leprosy treatment.

**Materials and Methods:**

Cross-sectional survey of 104 patients with leprosy followed in specialist reference centres in Sergipe, Brazil, between June and October 2014. QoL was evaluated using the World Health Organization-QoL-BREF (WHOQoL-BREF) questionnaire. The SALSA scale was used to measure FALs.

**Results:**

Low SALSA scores were present in 76% of patients. QoL scores were lower for the physical and environmental domains, with median (interquartile range (IQR)) scores of 53.6 (32.1–67.9) and 53.1 (46.9–64.8), respectively. There was a statistical association between increasing SALSA scores and lower QoL as measured by the WHOQoL-BREF.

**Conclusion:**

Functional limitations are associated with lower QoL in leprosy patients, especially in the physical and environmental WHOQoL-BREF domains.

## Author Summary

Leprosy is still a neglected public health problem. Leprosy causes disability and functional limitations (FALs) if not treated early. We describe the functional activity and quality of life (QoL) of adults with leprosy attending two reference centres in Sergipe, Brazil. Patients with leprosy had low QoL, which was associated to the degree of FALs. Despite increased access to diagnosis and modern multidrug leprosy therapy, leprosy is still strongly associated with FALs and low QoL. We stress here the importance of public health efforts to diagnose the disease early and to provide supportive systems for patients receiving treatment in endemic areas.

## Introduction

Leprosy is still a neglected public health problem with at least 200,000 new cases diagnosed annually worldwide. The highest prevalence occurs in low and middle income countries such as India, Brazil, Myanmar, Madagascar, Nepal, and Mozambique [1]. This chronic and insidious infection affects and impairs the skin and peripheral nerves and results in significant physical disability. This chronic and insidious infection affects and impairs the skin and peripheral nerves and results in significant physical disability. The clinical and pathological presentation of leprosy is determined by the immunological response to *Mycobacterium leprae* and the capacity of the host to develop an effective cell mediated immunity. In addition, leprosy-specific reactions are also a major cause of disability [2,3]. These reactions are called type 1 and 2, with type 1 essentially being a reversal reaction or ‘upgrading’ of the cell mediated immunity to *M*. *leprae* antibodies. These reactions are characterised by a marked increase in delayed type hypersensitivity and type 1 helper T lymphocyte cytokines. Type 2 reactions in turn are considered the result of immune complexes attracting granulocytes and complement activation with the selective activation of cytokines.

An estimated three million people exhibit leprosy-related impairments worldwide [4], generating severe social stigma and isolation, relationship and psychological problems and decreased ability to work [4,5].

The prevalence of disabilities due to leprosy varies among countries. Brazil has increased its detection of new cases with physical disability at diagnosis. In 2001, there was a 17.8% proportion of grade 1 physical disability and 6% of grade 2. In 2008, the proportion of grade 1 was 20.7% and grade 2 was 7.7% [6].

Functional activity limitations (FALs) associated with leprosy are well described. The main risk factors to develop FALs are the presence of leprosy reactions, presence of affected nerves, multibacillary leprosy and delay in diagnosis and/or treatment [7]. However, little is known about the interaction between these FALs and the quality of life (QoL). QoL is a broad concept including physical and psychological health, personal independence, social relationships, personal beliefs and the interaction of these factors with the environment. The term QoL incorporates the multidimensional nature and perception of overall quality of life but often is quoted as the impact of an illness or injury on the quality of life [8].

Currently, the main focus of rehabilitation centres is to prevent and treat physical impairment and to improve the QoL of patients. Therefore it is essential to assess how functional limitations affect the QoL of these patients to inform the development of interventions.

This study aimed to describe the relationship of FALs and the QoL of patients with a diagnosis of leprosy in an endemic area of Brazil.

## Materials and Methods

### Study design and patients

This was a cross-sectional survey to describe the FALs and QoL of patients attending two leprosy reference centres in Sergipe State, Northeast Brazil. Eligible criteria for inclusion in this study were patients >15 years old with diagnosis of leprosy and in MDT treatment or in treatment post-discharge for leprosy reactions. Patients with diabetes, excess alcohol consumption, known to be infected with the Human Immunodeficiency Virus, or with mental or physical conditions interfering with the assessment were excluded. All consecutive patients attending (a) the University Hospital Clinic and (b) the Leprosy and Tuberculosis Reference Centre, in Aracaju, Sergipe State, from June to October 2014 were enrolled.

### Questionnaires and procedures

After obtaining written informed consent to participate, participants were interviewed using a structured questionnaire that included demographic and clinical information (leprosy classification, leprosy reactions and disability grade) and an evaluation of their FALs and QoL. In addition, the clinical records of the patients and the database from the *Sistema de Informação de Agravos de Notificação* (SINAN) were reviewed to confirm the diagnosis and the presence of leprosy reactions. The SINAN is a national database containing information for all leprosy patients in Brazil.

Patients were classified using the World Health Organisation (WHO) leprosy classification as having paucibacillary (PB) (≤5 skin lesions and/or only one affected nerve trunk) or multibacillary (MB) leprosy (>5 skin lesions and/or >1 affected nerve trunk). Leprosy reactions were defined as episodes characterized by acute inflammation of skin lesions or nerves (type 1) and/or the appearance of inflamed cutaneous nodules with/without neuritis (type 2) [2]. The WHO disability classification was used. In this classification, grade 0 indicates no disability; grade 1 loss of sensibility in the eyes, hands and/or feet without visible deformity and grade 2 the loss of sensitivity and visible deformities [9].

QoL was assessed using the Portuguese version of the WHO-QoL-BREF (WHOQoL-BREF) questionnaire [10] (S1 and S2 Texts). The WHOQol-BREF is an international cross-culturally comparable QoL assessment instrument. This instrument is subdivided into physical, psychological, social relationships and environmental domains and each item is rated on a scale from 0 to 5, with higher scores indicating better QoL [8,11].

Functional Activity Limitations were measured using the Portuguese version of the Screening of Activity Limitation and Safety Awareness (SALSA) scale (S1 and S2 Texts). The scale measures activity limitations and risk awareness in patients who have or have had a disease with peripheral neuropathy, as in leprosy. The scale includes assessment of the eyes, hands (skills and labour), feet (mobility) and self-care. SALSA scores range from 10 to 80, with 10–24 allocated to patients without significant limitations; 25–39 for mild limitations and 40–49; 50–59 and 60–80 for moderate, severe and very severe limitations, respectively. The risk awareness score ranges from 0 to 11, with higher scores indicating greater awareness of the risks involved in daily life activities [7,12].

All questionnaires were completed by the interviewers in a quiet private place. The two interviewers were trained members of the team. We chose to interview the participants because patients with leprosy are often poor and have low educational level. When a respondent did not understand the meaning of a question, the interviewer re-read the question and did not explain the sentence with other words. The interviewers were not involved in the treatment of patients.

### Data analysis

Categorical variables were described using frequencies and percentages. The WHO analysis syntax for SPSS was used to calculate the four WHOQoL-BREF domain scores. Scores were standardised to a 4–20 scale, and domain scores were converted to a 0–100 scale as per the WHOQoL guidelines [11]. Pearson’s Chi-square or Fisher Exact Tests were used to compare the categorical variables association with the FALs. FALs were dichotomized as *present* (when there any limitation) or *absent* (without limitation). DG 1 and DG 2 were considered as disability for statistical analysis. The normal distribution of the scores was verified using the Kolmogorov-Smirnov test and most WHOQoL-BREF and SALSA scores had skewed distributions. We therefore used nonparametric tests to verify the significance of the distributions between the study variables. Kruskal-Wallis’s test was used to assess differences between measurements of the WHOQoL-BREF domains by the SALSA categories. When Kruskal-Wallis’s test was significant, we performed multiple comparisons using the Dunn's test (post-hoc test) to determinate differences between the groups. Spearman’s Rho correlations were used to describe the relationship between SALSA and WHOQoL-BREF domain scores. P values <5% were considered statistically significant.

### Ethics

The study was approved by the Human Research Ethics Committee of Federal University of Sergipe (CAAE: 31078114.3.0000.5546). All investigation has been conducted according to the Declaration of Helsinki. Informed consent written was obtained from the participants. Parents or guardians provided written informed consent before enrolling their children in the study.

## Results

One hundred and six patients were selected and invited to participate (S1 Table). Two patients who did not understand the WHOQoL-BREF questionnaire were excluded. Of the 104 patients included, 56 (53.8%) were male; their median (IQR) age was 48.0 (37.2–58.0) years old and the median (IQR) schooling was 5.0 (3–10) years. Twenty (19.3%) participants were receiving multidrug therapy (MDT) for leprosy at the time of the interview and 84 (80.7%) were receiving post-discharge treatment for leprosy reactions. There was no significant difference between the mean ages of patients receiving MDT or post-discharge treatment for leprosy reactions. Eighty-six (82.7%) participants had MB and 18 (17.3%) PB leprosy at the time of diagnosis. Twenty (19.2%) patients had leprosy-related deformities (Grade 2) (Table 1).

**Table 1:** Demographic characteristics of study participants.

Variables	N = 104
Sex, male, N (%)	56 (53.8%)
Age, median (IQR*) in years	48.0 (37.2–58.0)
Schooling, median (IQR*)	5.0 (3–10)
WHO leprosy classification, N (%)	
PB leprosy	86 (82.7%)
MB leprosy	18 (17.3%)
Leprosy reactions, N (%)	84 (80.7%)
WHO disability degree, N (%)	
DG0	29 (27.9%)
DG1	55 (52.9%)
DG2	20 (19.2%)
SALSA scale	
Functional limitation, N (%)	
Without limitation	25 (24.0%)
Mild	52 (50.0%)
Moderate	9 (8.7%)
Severe	6 (5.8%)
Very severe	12 (11.5%)
WHOQoL-BREF domains, median (IQR*)	
Physical	53.6 (32.1–67.9)
Psychological	62.5 (50.0–75.0)
Social	70.8 (58.3–75.0)
Environmental	53.1 (46.9–64.8)

*Interquartile range (IQR). Paucibacillary leprosy (PB). Multibacillary leprosy (MB). Disability Grade (DG). Screening of Activity Limitation and Safety Awareness scale (SALSA). World Health Organization-QoL-BREF (WHOQoL-BREF).

The median (interquartile range (IQR) SALSA score was 31.0 (25.0–41.5) points, with 25 (24%) patients having no significant FALs, 52 (50%) mild, 9 (8.7%) moderate, 6 (5.8%) severe and 12 (11.5) very severe FALs. There was an association between the presence of disabilities and FALs (p = 0.001). The median (IQR) SALSA score was higher in patients with MB leprosy than PB leprosy [33 (25.8–44.3) versus 25 (22.0–31.5), p = 0.02]. There was no difference in the SALSA score by sex (p = 0.10) or leprosy reaction (p = 0.20).

The median (IQR) scores for the WHOQoL-BREF domains were: 53.6 (32.1–67.9) for physical, 62.5 (50.0–75.0) for psychological, 70.8 (58.3–75.0) for social and 53.1 (46.9–64.8) for the environment domains (Table 1). Table 2 shows the WHOQoL-BREF scores by SALSA categories. There was a significant difference among the distribution of the SALSA categories into physical (χ^2^ = 45.6; p<0.001) and environmental (χ^2^ = 24.9; p<0.001) domains. Pairwise comparisons between SALSA categories were made using the Dunn’s test. Patients with moderate (0.001), severe (0.008) and very severe (<0.001) limitations had lower physical domain scores. Patients with severe (0.004) and very severe (0.001) limitations had lower environmental domain scores (Table 3).

**Table 2:** WHOQoL-BREF domain scores stratified by functional limitations.

Functional limitation	N (%)	WHOQoL-BREF domains Median (IQR*)			
		Physical	Psychological	Social	Environmental
Without limitation	25 (24.0)	71.4 (60.7–80.4)	70.8 (60.4–77.1)	75.0 (66.7–75.1)	62.5 (53.1–71.9)
Mild	52 (50.0)	56.6 (35.7–66.9)	62.5 (50.0–75.0)	75.0 (58.3–81.3)	53.1 (50.0–65.8)
Moderate	9 (8.7)	32.1 (30.4–46.4)	58.3 (50.0–75.0)	58.3 (45.8–79.2)	46.9 (43.8–59.4)
Severe	6 (5.8)	35.7 (25.0–51.8)	49.2 (29.2–69.8)	45.2 (12.5–87.5)	39.1 (35.2–45.3)
Very severe	12 (11.5)	25.0	54.2	58.3	45.3
p-value		p<0.001^a^	p = 0.20^a^	p = 0.34^a^	p<0.001^a^

^a^Kruskal-Wallis’s test.

* Interquartile range (IQR).

**Table 3:** Pairwise comparison of the SALSA categories by the physical and environmental domains.

Pairwise comparison	Physical domain p-value	Environmental domain p-value
Without and mild	0.018	0.75
Without and moderate	0.001	0.25
Without and severe	0.008	0.004
Without and very severe	<0.001	0.001

Increasing SALSA scores were associated with decreasing WHOQoL-BREF scores for the physical (r = -0.68; p<0.001), psychological (r = -0.28; p = 0.003), social (r = -0.21; p = 0.03) and environmental (r = -0.47; p<0.001) scores.

## Discussion

This study describes that patients with leprosy have FALs and that their presence, as assessed by SALSA, is associated with low QoL.

In Brazil, treatment and post-discharge follow-up of cases is routinely performed in primary health care centres and only cases with complications are referred to the centres of reference. Recent studies from Brazil of leprosy patients attending primary health care settings [7,13] have reported a FALs prevalence between 24% and 58% of cases and thus the higher prevalence observed is likely due to our participants being selected from reference centres.

Patients with leprosy had lower physical and environmental domain outcomes, which is in agreement with other Brazilian studies evaluating the QoL of people with leprosy sequelae [11,14]. In Bangladesh, patients undertaking leprosy treatment had lower scores in the psychological and physical domains but not in the environmental domain [15] and in India the lower values were reported in the social and environmental domains [16]. The differences between out study and the Asian reports therefore can be explained by the epidemiological context such as the characteristics of patients being enrolled in the studies and differences in the cultural and socioeconomic context, including the availability of long term rehabilitation and support programs.

Increased FAL was associated with decreased QoL in the four domains, with greater impairment in the physical and environmental domains. Deformities have multiple impacts on leprosy patients because they cause both functional limitations and a decreased perception of physical health. This is consistent with a study in India observing that physical domain scores were lower in deformed than in non-deformed patients [15].

The QoL scores in the environmental domain were associated with the presence of FALs with a strong inverse association between the prevalence of leprosy and income, education and social inequity [17]. Patients reported lower scores for ‘financial resources’, ‘information available’, ‘leisure activities’ and ‘satisfaction with transport’, which is consistent the socioeconomic level of most leprosy cases. Furthermore, the low scores in the environmental domain suggest difficulties in accessing healthcare services, despite diagnostics, care and treatment being available free of charge.

The psychological and social domains were inversely associated with FALs, as reported by others [4,5,15,18], although the Spearman Rho test showed a weak correlation between the FALs and these domains. Perhaps, the higher scores in the psychological and social domains than the physical and environment domains were due to the availability of supportive social networks and psychology services enhancing the capacity of patients to cope with the disease [11]. It is also important to note that we only included patients receiving treatment and those who had leprosy reactions. It would be important to document the FALs and QoL of patients who received leprosy treatment and have not experience leprosy reactions, as this group may have a very different QoL than the patients reported here.

The low QoL of patients with leprosy and leprosy reactions is associated with the presence of FALs, especially in the physical and environmental domains. Despite the increase in access to diagnosis and appropriate treatment using MDT in recent decades, patients with significant activity limitations may face reduced QoL. Fortunately, QoL and FALs can be improved by early treatment and rehabilitation interventions and functional limitations should be diagnosed early and monitored to assess the impact of treatment and rehabilitation.

## Supporting Information

S1 Checklist STROBE checklist. (DOC)

S1 Table Functional Limitation and Quality of Life in Leprosy database. (DOCX)

S1 Text English version of the WHO-QoL-BREF (WHOQoL-BREF) and Screening of Activity Limitation and Safety Awareness (SALSA) scale questionnaires. (DOC)

S2 Text Portuguese version of the WHO-QoL-BREF (WHOQoL-BREF) and Screening of Activity Limitation and Safety Awareness (SALSA) scale questionnaires. (DOCX)
