# Supplementary material for: Functional Activity Limitation and Quality of Life of Leprosy Cases in an Endemic Area in Northeastern Brazil
Source: PLoS Negl Trop Dis. 2015 Jul 1;9(7):e0003900. doi: 10.1371/journal.pntd.0003900 (PMC4489006; doi:10.1371/journal.pntd.0003900)
Supplement: S1 Table — (DOCX) [file pntd.0003900.s002.docx]

S1 Table. Functional Limitation and Quality of Life in Leprosy database.

| Id | Age | Sex | Schooling | Type treatment | WHO_Class | Disability Grade | PHYS_Score | PSYCH_Score | SOCIAL_Score | ENVIR_Score | SALSA_Score |
| --- | --- | --- | --- | --- | --- | --- | --- | --- | --- | --- | --- |
| 1 | 27 | Male | 11 | LR | MB | GRAU 0 | 67.86 | 87.5 | 91.67 | 87.5 | 18 |
| 2 | 19 | Female | 2 | MDT | PB | GRAU 0 | 75 | 45.83 | 66.67 | 53.13 | 23 |
| 3 | 46 | Male | 12 | LR | MB | GRAU 0 | 71.43 | 75 | 75 | 65.63 | 19 |
| 4 | 45 | Male | 0 | LR | PB | GRAU 0 | 50 | 70.83 | 75 | 62.5 | 23 |
| 5 | 55 | Male | 7 | MDT | PB | GRAU 0 | 60.71 | 54.17 | 75 | 46.88 | 19 |
| 6 | 29 | Female | 8 | LR | MB | GRAU 0 | 78.57 | 79.17 | 66.67 | 65.63 | 18 |
| 7 | 64 | Female | 15 | LR | PB | GRAU 0 | 60.71 | 62.5 | 75 | 53.13 | 22 |
| 8 | 60 | Male | 0 | MDT | MB | GRAU 0 | 82.14 | 70.83 | 75 | 75 | 22 |
| 9 | 19 | Female | 1 | MDT | MB | GRAU 0 | 71.43 | 50 | 66.67 | 53.13 | 19 |
| 10 | 39 | Female | 8 | LR | PB | GRAU 0 | 85.71 | 75 | 75 | 71.88 | 17 |
| 11 | 43 | Female | 8 | LR | PB | GRAU 0 | 57.14 | 66.67 | 75 | 62.5 | 22 |
| 12 | 18 | Female | 5 | LR | MB | GRAU 0 | 85.71 | 75 | 100 | 87.5 | 24 |
| 13 | 33 | Male | 4 | LR | PB | GRAU 0 | 89.29 | 95 | 100 | 53.13 | 20 |
| 14 | 52 | Female | 3 | LR | PB | GRAU 0 | 21.43 | 33.33 | 16.67 | 34.38 | 24 |
| 15 | 71 | Male | 7 | LR | MB | GRAU 2 | 46.43 | 58.33 | 50 | 46.88 | 23 |
| 16 | 74 | Female | 0 | MDT | MB | GRAU 2 | 60.71 | 62.5 | 75 | 71.88 | 21 |
| 17 | 69 | Male | 6 | LR | MB | GRAU 2 | 85.71 | 83.33 | 75 | 75 | 20 |
| 18 | 43 | Male | 8 | MDT | MB | GRAU 1 | 78.57 | 66.67 | 58.33 | 59.38 | 19 |
| 19 | 52 | Female | 4 | LR | MB | GRAU 1 | 64.29 | 20.83 | 41.67 | 43.75 | 22 |
| 20 | 53 | Male | 15 | LR | MB | GRAU 2 | 67.86 | 79.17 | 75 | 68.75 | 15 |
| 21 | 60 | Female | 6 | LR | MB | GRAU 1 | 64.29 | 62.5 | 75 | 62.5 | 24 |
| 22 | 69 | Male | 8 | LR | MB | GRAU 1 | 82.14 | 83.33 | 66.67 | 65.63 | 20 |
| 23 | 71 | Male | 4 | MDT | MB | GRAU 1 | 78.57 | 75 | 66.67 | 59.38 | 22 |
| 24 | 63 | Female | 0 | LR | MB | GRAU 1 | 75 | 70.83 | 66.67 | 68.75 | 22 |
| 25 | 21 | Female | 0 | LR | MB | GRAU 1 | 75 | 66.67 | 58.33 | 71.88 | 24 |
| 26 | 50 | Male | 11 | LR | MB | GRAU 1 | 60.71 | 66.67 | 25 | 53.13 | 33 |
| 27 | 27 | Male | 9 | LR | PB | GRAU 1 | 96.43 | 79.17 | 91.67 | 81.25 | 35 |
| 28 | 31 | Male | 0 | LR | MB | GRAU 1 | 64.29 | 87.5 | 100 | 75 | 28 |
| 29 | 39 | Male | 4 | LR | MB | GRAU 1 | 50 | 75 | 66.67 | 53.13 | 33 |
| 30 | 48 | Male | 11 | LR | MB | GRAU 1 | 92.86 | 83.33 | 91.67 | 75 | 34 |
| 31 | 45 | Female | 4 | LR | MB | GRAU 1 | 57.14 | 75 | 75 | 62.5 | 30 |
| 32 | 48 | Female | 4 | LR | MB | GRAU 1 | 75 | 79.17 | 83.33 | 71.88 | 28 |
| 33 | 50 | Male | 0 | MDT | MB | GRAU 1 | 42.86 | 66.67 | 91.67 | 56.25 | 37 |
| 34 | 59 | Male | 3 | LR | MB | GRAU 1 | 57.14 | 83.33 | 83.33 | 53.13 | 38 |
| 35 | 41 | Male | 0 | LR | MB | GRAU 1 | 35.71 | 50 | 75 | 53.13 | 33 |
| 36 | 48 | Female | 0 | LR | MB | GRAU 1 | 78.57 | 79.17 | 66.67 | 59.38 | 31 |
| 37 | 78 | Female | 6 | LR | MB | GRAU 1 | 67.86 | 79.17 | 91.67 | 71.88 | 35 |
| 38 | 34 | Female | 4 | LR | MB | GRAU 1 | 53.57 | 62.5 | 83.33 | 56.25 | 31 |
| 39 | 35 | Female | 5 | LR | MB | GRAU 0 | 53.57 | 62.5 | 75 | 62.5 | 31 |
| 40 | 29 | Male | 7 | LR | MB | GRAU 0 | 42.86 | 58.33 | 50 | 56.25 | 29 |
| 41 | 33 | Male | 11 | LR | PB | GRAU 0 | 50 | 45.83 | 75 | 50 | 25 |
| 42 | 84 | Male | 8 | LR | MB | GRAU 0 | 42.86 | 58.33 | 50 | 46.88 | 33 |
| 43 | 41 | Male | 15 | LR | MB | GRAU 0 | 35.71 | 41.67 | 66.67 | 53.13 | 34 |
| 44 | 54 | Female | 8 | MDT | PB | GRAU 0 | 71.43 | 62.5 | 75 | 53.13 | 28 |
| 45 | 53 | Male | 3 | LR | MB | GRAU 0 | 64.29 | 62.5 | 66.67 | 56.25 | 25 |
| 46 | 42 | Female | 4 | MDT | MB | GRAU 0 | 35.71 | 58.33 | 66.67 | 56.25 | 35 |
| 47 | 56 | Female | 10 | LR | MB | GRAU 0 | 35.71 | 70.83 | 75 | 53.13 | 28 |
| 48 | 39 | Female | 5 | LR | MB | GRAU 0 | 46.43 | 62.5 | 33.33 | 46.88 | 26 |
| 49 | 52 | Female | 17 | LR | MB | GRAU 0 | 28.57 | 16.67 | 25 | 37.5 | 34 |
| 50 | 74 | Female | 11 | LR | PB | GRAU 0 | 85.71 | 91.67 | 91.67 | 68.75 | 25 |
| 51 | 41 | Male | 5 | LR | MB | GRAU 0 | 46.43 | 62.5 | 75 | 37.5 | 36 |
| 52 | 47 | Male | 11 | LR | MB | GRAU 2 | 17.86 | 37.5 | 41.67 | 50 | 39 |
| 53 | 58 | Male | 0 | MDT | MB | GRAU 2 | 60.71 | 25 | 50 | 90.63 | 28 |
| 54 | 64 | Male | 11 | MDT | MB | GRAU 2 | 78.57 | 87.5 | 66.67 | 62.5 | 30 |
| 55 | 62 | Female | 13 | LR | MB | GRAU 2 | 64.29 | 75 | 66.67 | 65.63 | 26 |
| 56 | 37 | Male | 0 | MDT | MB | GRAU 2 | 28.57 | 66.67 | 75 | 43.75 | 32 |
| 57 | 63 | Female | 0 | LR | MB | GRAU 1 | 60.71 | 66.67 | 75 | 71.88 | 29 |
| 58 | 63 | Male | 0 | LR | MB | GRAU 1 | 25 | 37.5 | 50 | 53.13 | 34 |
| 59 | 48 | Male | 11 | LR | MB | GRAU 1 | 25 | 25 | 58.33 | 50 | 27 |
| 60 | 54 | Male | 0 | MDT | MB | GRAU 1 | 53.57 | 62.5 | 75 | 46.88 | 25 |
| 61 | 61 | Male | 0 | LR | MB | GRAU 2 | 17.86 | 50 | 41.67 | 53.13 | 33 |
| 62 | 63 | Female | 8 | LR | MB | GRAU 1 | 64.29 | 66.67 | 75 | 59.38 | 27 |
| 63 | 50 | Female | 5 | LR | MB | GRAU 2 | 25 | 41.67 | 33.33 | 46.88 | 38 |
| 64 | 50 | Female | 4 | MDT | MB | GRAU 2 | 35.71 | 75 | 75 | 50 | 25 |
| 65 | 74 | Female | 0 | LR | MB | GRAU 1 | 60.71 | 83.33 | 75 | 62.5 | 28 |
| 66 | 79 | Female | 14 | LR | PB | GRAU 1 | 75 | 70.83 | 66.67 | 56.25 | 30 |
| 67 | 38 | Female | 1 | LR | MB | GRAU 2 | 39.29 | 12.5 | 8.33 | 31.25 | 29 |
| 68 | 52 | Male | 0 | LR | PB | GRAU 1 | 71.43 | 62.5 | 75 | 71.88 | 31 |
| 69 | 49 | Male | 8 | LR | MB | GRAU 1 | 67.86 | 83.33 | 83.33 | 40.63 | 25 |
| 70 | 34 | Female | 8 | LR | MB | GRAU 1 | 82.14 | 91.67 | 91.67 | 50 | 29 |
| 71 | 27 | Female | 5 | LR | MB | GRAU 1 | 32.14 | 29.17 | 33.33 | 37.5 | 34 |
| 72 | 29 | Female | 3 | LR | PB | GRAU 1 | 50 | 75 | 91.67 | 68.75 | 33 |
| 73 | 56 | Female | 14 | LR | MB | GRAU 1 | 67.86 | 66.67 | 83.33 | 84.38 | 34 |
| 74 | 23 | Female | 4 | LR | PB | GRAU 1 | 57.14 | 62.5 | 75 | 65.63 | 31 |
| 75 | 30 | Female | 0 | LR | MB | GRAU 1 | 53.57 | 50 | 58.33 | 53.13 | 36 |
| 76 | 45 | Male | 0 | LR | MB | GRAU 1 | 57.14 | 45.83 | 58.33 | 50 | 37 |
| 77 | 50 | Female | 4 | LR | MB | GRAU 2 | 35.71 | 58.33 | 75 | 43.75 | 34 |
| 78 | 51 | Female | 5 | MDT | MB | GRAU 1 | 14.29 | 41.67 | 75 | 46.88 | 42 |
| 79 | 67 | Male | 0 | MDT | MB | GRAU 1 | 32.14 | 79.17 | 83.33 | 59.38 | 48 |
| 80 | 49 | Female | 3 | LR | MB | GRAU 1 | 32.14 | 70.83 | 41.67 | 46.88 | 49 |
| 81 | 21 | Male | 10 | LR | MB | GRAU 1 | 50 | 58.33 | 58.33 | 46.88 | 48 |
| 82 | 54 | Male | 2 | LR | MB | GRAU 1 | 28.57 | 58.33 | 25 | 31.25 | 48 |
| 83 | 36 | Male | 4 | MDT | MB | GRAU 2 | 35.71 | 41.67 | 58.33 | 53.13 | 44 |
| 84 | 69 | Female | 0 | LR | MB | GRAU 1 | 42.86 | 66.67 | 75 | 59.38 | 40 |
| 85 | 55 | Male | 10 | LR | MB | GRAU 2 | 32.14 | 58.33 | 50 | 40.63 | 45 |
| 86 | 41 | Male | 8 | LR | MB | GRAU 2 | 64.29 | 87.5 | 91.67 | 75 | 43 |
| 87 | 37 | Male | 3 | LR | MB | GRAU 1 | 57.14 | 66.67 | 83.33 | 59.38 | 58 |
| 88 | 44 | Male | 3 | LR | MB | GRAU 0 | 39.29 | 40 | 16.67 | 40.63 | 50 |
| 89 | 50 | Male | 0 | LR | MB | GRAU 0 | 14.29 | 16.67 | 0 | 37.5 | 55 |
| 90 | 46 | Female | 7 | LR | MB | GRAU 1 | 32.14 | 79.17 | 100 | 28.13 | 57 |
| 91 | 37 | Male | 10 | MDT | MB | GRAU 2 | 28.57 | 58.33 | 66.67 | 40.63 | 58 |
| 92 | 45 | Male | 0 | LR | MB | GRAU 1 | 50 | 33.33 | 41.67 | 37.5 | 50 |
| 93 | 43 | Male | 14 | LR | MB | GRAU 1 | 14.29 | 75 | 50 | 50 | 80 |
| 94 | 37 | Male | 3 | LR | MB | GRAU 1 | 32.14 | 62.5 | 50 | 0 | 66 |
| 95 | 32 | Male | 0 | LR | MB | GRAU 1 | 25 | 33.33 | 41.67 | 50 | 64 |
| 96 | 43 | Male | 9 | LR | MB | GRAU 1 | 25 | 66.67 | 75 | 59.38 | 67 |
| 97 | 37 | Female | 0 | LR | MB | GRAU 1 | 32.14 | 58.33 | 58.33 | 43.75 | 74 |
| 98 | 53 | Male | 5 | LR | PB | GRAU 1 | 14.29 | 45.83 | 83.33 | 37.5 | 63 |
| 99 | 58 | Male | 3 | LR | MB | GRAU 1 | 25 | 75 | 58.33 | 46.88 | 68 |
| 100 | 48 | Female | 11 | MDT | PB | GRAU 1 | 17.86 | 20.83 | 58.33 | 31.25 | 78 |
| 101 | 60 | Male | 14 | LR | MB | GRAU 1 | 21.43 | 50 | 41.67 | 37.5 | 74 |
| 102 | 39 | Female | 0 | LR | MB | GRAU 1 | 28.57 | 41.67 | 66.67 | 40.63 | 64 |
| 103 | 71 | Male | 14 | LR | MB | GRAU 2 | 25 | 87.5 | 66.67 | 46.88 | 69 |
| 104 | 52 | Female | 7 | LR | MB | GRAU 2 | 17.86 | 37.5 | 75 | 56.25 | 80 |
