# Supplementary material for: Functional Activity Limitation and Quality of Life of Leprosy Cases in an Endemic Area in Northeastern Brazil
Source: PLoS Negl Trop Dis. 2015 Jul 1;9(7):e0003900. doi: 10.1371/journal.pntd.0003900 (PMC4489006; doi:10.1371/journal.pntd.0003900)
Supplement: S1 Text — (DOC) [file pntd.0003900.s003.doc]

**S1 Appendix**

**English version of the WHO-QoL-BREF (WHOQoL-BREF) questionnaire**

The following questions ask how you feel about your quality of life, health, or other areas of your life. I will read out each question to you, along with the response options. **Please** **choose** **the** **answer** **that** **appears** **most** **appropriate.** If you are unsure about which response to give to a question, the first response you think of is often the best one.

Please keep in mind your standards, hopes, pleasures and concerns. We ask that you think about your life **in** **the** **last** **four** **weeks.**

|  |  | Very poor | Poor | Neither poor nor good | Good | Very good |
| --- | --- | --- | --- | --- | --- | --- |
| 1. | How would you rate your quality of life? | 1 | 2 | 3 | 4 | 5 |

|  |  | Very dissatisfied | Dissatisfied | Neither satisfied nor dissatisfied | Satisfied | Very satisfied |
| --- | --- | --- | --- | --- | --- | --- |
| 2. | How satisfied are you with your health? | 1 | 2 | 3 | 4 | 5 |

The following questions ask about **how** **much** you have experienced certain things in the last four weeks.

|  |  | Not at all | A little | A moderate amount | Very much | An extreme amount |
| --- | --- | --- | --- | --- | --- | --- |
| 3. | To what extent do you feel that physical pain prevents you from  doing what you need to do? | 5 | 4 | 3 | 2 | 1 |
| 4. | How much do you need any medical treatment to function in your daily life? | 5 | 4 | 3 | 2 | 1 |
| 5. | How much do you enjoy life? | 1 | 2 | 3 | 4 | 5 |
| 6. | To what extent do you feel your life to be meaningful? | 1 | 2 | 3 | 4 | 5 |

|  |  | Not at all | A little | A moderate amount | Very much | Extremely |
| --- | --- | --- | --- | --- | --- | --- |
| 7. | How well are you able to concentrate? | 1 | 2 | 3 | 4 | 5 |
| 8. | How safe do you feel in your daily life? | 1 | 2 | 3 | 4 | 5 |
| 9. | How healthy is your physical environment? | 1 | 2 | 3 | 4 | 5 |

The following questions ask about how completely you experience or were able to do certain things in the last four weeks.

|  |  | Not at all | A little | Moderately | Mostly | Completely |
| --- | --- | --- | --- | --- | --- | --- |
| 10. | Do you have enough energy for everyday life? | 1 | 2 | 3 | 4 | 5 |
| 11. | Are you able to accept your bodily appearance? | 1 | 2 | 3 | 4 | 5 |
| 12. | Have you enough money to meet your needs? | 1 | 2 | 3 | 4 | 5 |
| 13. | How available to you is the information that you need in your day-to-day life? | 1 | 2 | 3 | 4 | 5 |
| 14. | To what extent do you have the opportunity for leisure activities? | 1 | 2 | 3 | 4 | 5 |

|  |  | Very poor | Poor | Neither poor nor good | Good | Very good |
| --- | --- | --- | --- | --- | --- | --- |
| 15. | How well are you able to get around? | 1 | 2 | 3 | 4 | 5 |

|  |  | Very dissatisfied | Dissatisfied | Neither satisfied nor dissatisfied | Satisfied | Very satisfied |
| --- | --- | --- | --- | --- | --- | --- |
| 16. | How satisfied are you with your sleep? | 1 | 2 | 3 | 4 | 5 |
| 17. | How satisfied are you with your ability to perform your daily living activities? | 1 | 2 | 3 | 4 | 5 |
| 18. | How satisfied are you with your capacity for work? | 1 | 2 | 3 | 4 | 5 |
| 19. | How satisfied are you with yourself? | 1 | 2 | 3 | 4 | 5 |

| 20. | How satisfied are you with your personal relationships? | 1 | 2 | 3 | 4 | 5 |
| --- | --- | --- | --- | --- | --- | --- |
| 21. | How satisfied are you with your sex life? | 1 | 2 | 3 | 4 | 5 |
| 22. | How satisfied are you with the support you get from your friends? | 1 | 2 | 3 | 4 | 5 |
| 23. | How satisfied are you with the conditions of your living place? | 1 | 2 | 3 | 4 | 5 |
| 24. | How satisfied are you with your access to health services? | 1 | 2 | 3 | 4 | 5 |
| 25. | How satisfied are you with your transport? | 1 | 2 | 3 | 4 | 5 |

The following question refers to how often you have felt or experienced certain things in the last four weeks.

|  |  | Never | Seldom | Quite often | Very often | Always |
| --- | --- | --- | --- | --- | --- | --- |
| 26. | How often do you have negative feelings such as blue mood, despair, anxiety, depression? | 5 | 4 | 3 | 2 | 1 |

**English version of the Screening of Activity Limitation and Safety Awareness (SALSA) scale**

|  | **Domains** | **SALSA scale**  **S**creening of **A**ctivity **L**imitation & **S**afety **A**wareness  **Tick one box on each line in response to each question** | **If Yes, how easy is it for you?** | | | **If No, why not?** | | |
| --- | --- | --- | --- | --- | --- | --- | --- | --- |
|  |  |  | **Easy** | **Little difficult** | **Very difficult** | **I don’t need to do this** | **I physically cannot** | **I avoid because of risk** |
| 1. |  | **Can you see** (enough to carry out your daily activities)**?** | 1 | 2 | 3 |  | 4 |  |
| 2. | **Mobility (feet)** | **Do you sit or squat on the ground?** | 1 | 2 | 3 | 0 | 4 | 4 |
| 3. |  | **Do you walk barefoot?** e.g. most of the time | 1 | 2 | 3 | 0 |  |  |
| 4. |  | **Do you walk on uneven ground?** | 1 | 2 | 3 | 0 |  |  |
| 5. |  | **Do you walk longer distances?** i.e. longer than 30 minutes | 1 | 2 | 3 | 0 |  |  |
| 6. | **Self care** | **Do you wash your whole body?** (using soap, sponge, jug; standing or sitting) | 1 | 2 | 3 | 0 | 4 | 4 |
| 7. |  | **Do you cut your finger or toenails?** e.g. using scissors or clippers… | 1 | 2 | 3 | 0 |  |  |
| 8. |  | **Do you hold a cup or basin with hot contents?** e.g. drinks, food… | 1 | 2 | 3 | 0 | 4 | 4 |
| 9. | **Work (hands)** | **Do you work with tools?** i.e. tools which you hold in your hands to help you work… | 1 | 2 | 3 | 0 |  |  |
| 10. |  | **Do you carry heavy objects or bags?** e.g. shopping, food, water, wood… | 1 | 2 | 3 | 0 |  |  |
| 11. |  | **Do you lift objects above your head?** e.g. to place on a shelf, on your head, to hang clothes to dry... | 1 | 2 | 3 | 0 |  |  |
| 12. |  | **Do you cook?** i.e. prepare food both hot and cold | 1 | 2 | 3 | 0 |  |  |
| 13. |  | **Do you pour hot liquids?** | 1 | 2 | 3 | 0 |  |  |
| 14. |  | **Do you open/close screw capped bottles?** e.g. oil, water… | 1 | 2 | 3 | 0 | 4 | 4 |
| 15. |  | **Do you open jars with screw-on lids?** e.g. jam… | 1 | 2 | 3 | 0 |  |  |
| 16. | **Dexterity (hands)** | **Do you handle or manipulate small objects?** e.g. coins, nails, small screws, grains and seeds… | 1 | 2 | 3 | 0 | 4 | 4 |
| 17. |  | **Do you use buttons?** e.g. buttons on clothing, bags… | 1 | 2 | 3 | 0 | 4 | 4 |
| 18. |  | **Do you thread needles?** i.e. pass thread through the eye of a needle | 1 | 2 | 3 | 0 |  |  |
| 19. |  | **Do you pick up pieces of paper, handle paper or put it in order?** | 1 | 2 | 3 | 0 | 4 | 4 |
| 20. |  | **Do you pick up things from the floor?** | 1 | 2 | 3 | 0 | 4 | 4 |
|  |  | Sub total scores | (S1) | (S2) | (S3) | (S4) | (S5) | (S6) |
|  |  | SALSA score (*add up all sub total score*) | (S1+S2+S3+S4+S5+S6) | | | | | |
|  |  | Safety Awareness score ( *Count the number of ticked 4’s in each column*) | | | | |  |  |
