# Supplementary material for: Functional Activity Limitation and Quality of Life of Leprosy Cases in an Endemic Area in Northeastern Brazil
Source: PLoS Negl Trop Dis. 2015 Jul 1;9(7):e0003900. doi: 10.1371/journal.pntd.0003900 (PMC4489006; doi:10.1371/journal.pntd.0003900)
Supplement: S2 Text — (DOCX) [file pntd.0003900.s004.docx]

**S2 Appendix**

**Portuguese version of the WHO-QoL-BREF (WHOQoL-BREF) questionnaire**

1- Como você avaliaria sua qualidade de vida?

| Muito ruim | Ruim | Nem ruim  Nem boa | Boa | Muito boa |
| --- | --- | --- | --- | --- |
| 1 | 2 | 3 | 4 | 5 |

2- Quão satisfeito (a) você está com a sua saúde?

| Muito insatisfeito | Insatisfeito | Nem satisfeito  Nem insatisfeito | Satisfeito | Muito satisfeito |
| --- | --- | --- | --- | --- |
| 1 | 2 | 3 | 4 | 5 |

- As questões seguintes são sobre o quanto você tem sentido algumas coisas nas últimas semanas:

3- Em que medida você acha que sua dor (física) impede você de fazer o que você precisa?

| Nada | Muito pouco | Mais ou menos | Bastante | Extremamente |
| --- | --- | --- | --- | --- |
| 1 | 2 | 3 | 4 | 5 |

4- O quanto você precisa de algum tratamento médico para levar sua vida diária?

| Nada | Muito pouco | Mais ou menos | Bastante | Extremamente |
| --- | --- | --- | --- | --- |
| 1 | 2 | 3 | 4 | 5 |

5- O quanto você aproveita a vida?

| Nada | Muito pouco | Mais ou menos | Bastante | Extremamente |
| --- | --- | --- | --- | --- |
| 1 | 2 | 3 | 4 | 5 |

6- Em que medida você acha que a sua vida tem sentido?

| Nada | Muito pouco | Mais ou menos | Bastante | Extremamente |
| --- | --- | --- | --- | --- |
| 1 | 2 | 3 | 4 | 5 |

7- O quanto você consegue se concentrar?

| Nada | Muito pouco | Mais ou menos | Bastante | Extremamente |
| --- | --- | --- | --- | --- |
| 1 | 2 | 3 | 4 | 5 |

8- Quão seguro(a) você se sente em sua vida diária?

| Nada | Muito pouco | Mais ou menos | Bastante | Extremamente |
| --- | --- | --- | --- | --- |
| 1 | 2 | 3 | 4 | 5 |

9- Quão saudável é o seu ambiente físico (clima, barulho, poluição, atrativos)?

| Nada | Muito pouco | Mais ou menos | Bastante | Extremamente |
| --- | --- | --- | --- | --- |
| 1 | 2 | 3 | 4 | 5 |

- As questões seguintes perguntam sobre quão completamente você tem sentido ou é capaz de fazer certas coisas nestas últimas duas semanas.

10- Você tem energia suficiente para seu dia-a-dia?

| Nada | Muito pouco | Médio | Muito | Completamente |
| --- | --- | --- | --- | --- |
| 1 | 2 | 3 | 4 | 5 |

11- Você é capaz de aceitar sua aparência física?

| Nada | Muito pouco | Médio | Muito | Completamente |
| --- | --- | --- | --- | --- |
| 1 | 2 | 3 | 4 | 5 |

12- Você tem dinheiro suficiente para satisfazer suas necessidades?

| Nada | Muito pouco | Médio | Muito | Completamente |
| --- | --- | --- | --- | --- |
| 1 | 2 | 3 | 4 | 5 |

13- Quão disponíveis para você estão as informações que precisa no seu dia-a-dia?

| Nada | Muito pouco | Médio | Muito | Completamente |
| --- | --- | --- | --- | --- |
| 1 | 2 | 3 | 4 | 5 |

14- Em que medida você tem oportunidades de atividade de lazer?

| Nada | Muito pouco | Médio | Muito | Completamente |
| --- | --- | --- | --- | --- |
| 1 | 2 | 3 | 4 | 5 |

- As questões seguintes perguntam sobre quão bem ou satisfeito você se sentiu a respeito de vários aspectos de sua vida nas últimas duas semanas.

15- Quão bem você é capaz de se locomover?

| Muito ruim | Ruim | Nem ruim  Nem bom | Bom | Muito bom |
| --- | --- | --- | --- | --- |
| 1 | 2 | 3 | 4 | 5 |

16- Quão satisfeito(a) você está com o seu sono?

| Muito insatisfeito | Insatisfeito | Nem satisfeito  Nem insatisfeito | Satisfeito | Muito satisfeito |
| --- | --- | --- | --- | --- |
| 1 | 2 | 3 | 4 | 5 |

17- Quão satisfeito(a) você está com sua capacidade de desempenhar as atividades do seu dia-a-dia?

| Muito insatisfeito | Insatisfeito | Nem satisfeito  Nem insatisfeito | Satisfeito | Muito satisfeito |
| --- | --- | --- | --- | --- |
| 1 | 2 | 3 | 4 | 5 |

18- Quão satisfeito(a) você está com sua capacidade para o trabalho?

| Muito insatisfeito | Insatisfeito | Nem satisfeito  Nem insatisfeito | Satisfeito | Muito satisfeito |
| --- | --- | --- | --- | --- |
| 1 | 2 | 3 | 4 | 5 |

19- Quão satisfeito(a) você está consigo mesmo?

| Muito insatisfeito | Insatisfeito | Nem satisfeito  Nem insatisfeito | Satisfeito | Muito satisfeito |
| --- | --- | --- | --- | --- |
| 1 | 2 | 3 | 4 | 5 |

20- Quão satisfeito(a) você está com suas relações pessoais (amigos, parentes, conhecidos, colegas)?

| Muito insatisfeito | Insatisfeito | Nem satisfeito  Nem insatisfeito | Satisfeito | Muito satisfeito |
| --- | --- | --- | --- | --- |
| 1 | 2 | 3 | 4 | 5 |

21- Quão satisfeito(a) você está com sua vida sexual?

| Muito insatisfeito | Insatisfeito | Nem satisfeito  Nem insatisfeito | Satisfeito | Muito satisfeito |
| --- | --- | --- | --- | --- |
| 1 | 2 | 3 | 4 | 5 |

22- Quão satisfeito(a) você está com o apoio que você recebe de seus amigos?

| Muito insatisfeito | Insatisfeito | Nem satisfeito  Nem insatisfeito | Satisfeito | Muito satisfeito |
| --- | --- | --- | --- | --- |
| 1 | 2 | 3 | 4 | 5 |

23- Quão satisfeito(a) você está com as condições do local onde mora?

| Muito insatisfeito | Insatisfeito | Nem satisfeito  Nem insatisfeito | Satisfeito | Muito satisfeito |
| --- | --- | --- | --- | --- |
| 1 | 2 | 3 | 4 | 5 |

24- Quão satisfeito(a) você está com o seu acesso aos serviços de saúde?

| Muito insatisfeito | Insatisfeito | Nem satisfeito  Nem insatisfeito | Satisfeito | Muito satisfeito |
| --- | --- | --- | --- | --- |
| 1 | 2 | 3 | 4 | 5 |

25- Quão satisfeito(a) você está com o seu meio de transporte?

| Muito insatisfeito | Insatisfeito | Nem satisfeito  Nem insatisfeito | Satisfeito | Muito satisfeito |
| --- | --- | --- | --- | --- |
| 1 | 2 | 3 | 4 | 5 |

- As questões seguintes referem-se a com que frequência você sentiu ou experimentou certas coisas nas últimas duas semanas.

26- Com que frequência você tem sentimentos negativos tais como mau humor, desespero, ansiedade, depressão?

| Nunca | Algumas vezes | Frequentemente | Muito frequentemente | Sempre |
| --- | --- | --- | --- | --- |
| 1 | 2 | 3 | 4 | 5 |

**Portuguese version of the Screening of Activity Limitation and Safety Awareness (SALSA) scale**

|  | **Domínios** | **Escala SALSA**  **S**creening of **A**ctivity **L**imitation & **S**afety **A**wareness  (Triagem de Limitação de Atividade e Consciência de Risco)  **Marque uma resposta em cada linha** | **Se SIM, o quanto isso é fácil para você?** | | | **Se NÃO, por que não?** | | |
| --- | --- | --- | --- | --- | --- | --- | --- | --- |
|  |  |  | **Fácil** | **Um pouco difícil** | **Muito difícil** | **Eu não preciso fazer isso** | **Eu fisicamente não consigo** | **Eu evito por causa do risco** |
| 1. |  | **Você consegue enxergar** (o suficiente para realizar suas atividades diárias)**?** | 1 | 2 | 3 |  | 4 |  |
| 2. | **Mobilidade (pés)** | **Você se senta ou agacha no chão?** | 1 | 2 | 3 | 0 | 4 | 4 |
| 3. |  | **Você anda descalço?** i.e. a maior parte do tempo | 1 | 2 | 3 | 0 |  |  |
| 4. |  | **Você anda sobre chão irregular?** | 1 | 2 | 3 | 0 |  |  |
| 5. |  | **Você anda distâncias mais longas?** i.e. mais que 30 minutos | 1 | 2 | 3 | 0 |  |  |
| 6. | **Auto cuidado** | **Você lava seu corpo todo?** (usando sabão, esponja, jarra; de pé ou sentado) | 1 | 2 | 3 | 0 | 4 | 4 |
| 7. |  | **Você corta as unhas das mãos ou dos pés?** e.g. usando tesoura ou cortador | 1 | 2 | 3 | 0 |  |  |
| 8. |  | **Você segura um copo/tigela com conteúdo quente?** e.g. bebida, comida | 1 | 2 | 3 | 0 | 4 | 4 |
| 9. | **Trabalho (mãos)** | **Você trabalha com ferramentas?** i.e. ferramentas que você segura com as mãos para ajudar a trabalhar | 1 | 2 | 3 | 0 |  |  |
| 10. |  | **Você carrega objetos ou sacolas pesadas?** e.g. compras, comida, água, lenha | 1 | 2 | 3 | 0 |  |  |
| 11. |  | **Você levanta objetos acima de sua cabeça?** e.g. para colocar em uma prateleira, em cima de sua cabeça, para estender roupa para secar | 1 | 2 | 3 | 0 |  |  |
| 12. |  | **Você cozinha?** i.e. preparar comida quente ou fria | 1 | 2 | 3 | 0 |  |  |
| 13. |  | **Você despeja/serve líquidos quentes?** | 1 | 2 | 3 | 0 |  |  |
| 14. |  | **Você abre/fecha garrafas com tampa de rosca?** e.g. óleo, água | 1 | 2 | 3 | 0 | 4 | 4 |
| 15. |  | **Você abre vidros com tampa de rosca?** e.g. maionese | 1 | 2 | 3 | 0 |  |  |
| 16. | **Destreza (mãos)** | **Você mexe/manipula objetos pequenos?** e.g. moedas, pregos, parafusos pequenos, grãos, sementes | 1 | 2 | 3 | 0 | 4 | 4 |
| 17. |  | **Você usa botões?** e.g. botões em roupas, bolsas | 1 | 2 | 3 | 0 | 4 | 4 |
| 18. |  | **Você coloca linha na agulha?** i.e. passa a linha pelo olho da agulha | 1 | 2 | 3 | 0 |  |  |
| 19. |  | **Você apanha pedaços de papel, mexe com papel/coloca papel em ordem?** | 1 | 2 | 3 | 0 | 4 | 4 |
| 20. |  | **Você apanha coisas do chão?** | 1 | 2 | 3 | 0 | 4 | 4 |
|  |  | Escores parciais | (S1) | (S2) | (S3) | (S4) | (S5) | (S6) |
|  |  | Escore SALSA (*some todos os escores parciais*) | (S1+S2+S3+S4+S5+S6) | | | | | |
|  |  | Escore de consciência de risco (*Conte o número de ’s marcados em cada coluna*) | | | | |  |  |
